# COVID-19 and influenza vaccine uptake among pregnant women in national cohorts of England and Wales

**DOI:** 10.1038/s41541-024-00934-9

**Published:** 2024-08-14

**Authors:** Xinchun Gu, Utkarsh Agrawal, William Midgley, Stuart Bedston, Sneha N. Anand, Rosalind Goudie, Rachel Byford, Mark Joy, Gavin Jamie, Uy Hoang, Jose M. Ordóñez-Mena, Chris Robertson, F. D. Richard Hobbs, Ashley Akbari, Aziz Sheikh, Simon de Lusignan

**Affiliations:** 1 Nuffield Department of Primary Care Health Sciences, University of Oxford, Oxford, UK; 2 Population Data Science, Swansea University Medical School, Swansea University, Swansea, UK; 3 University of Strathclyde and Public Health Scotland, Glasgow, UK; 4 Usher Institute, The University of Edinburgh, Edinburgh, UK

**Keywords:** Epidemiology, Risk factors

## Abstract

Vaccines against COVID-19 and influenza can reduce the adverse outcomes caused by infections during pregnancy, but vaccine uptake among pregnant women has been suboptimal. We examined the COVID-19 and influenza vaccine uptake and disparities in pregnant women during the COVID-19 pandemic to inform vaccination interventions. We used data from the Oxford-Royal College of General Practitioners Research and Surveillance Centre database in England and the Secure Anonymised Information Linkage Databank in Wales. The uptake of at least one dose of vaccine was 40.2% for COVID-19 and 41.8% for influenza among eligible pregnant women. We observed disparities in COVID-19 and influenza vaccine uptake, with socioeconomically deprived and ethnic minority groups showing lower vaccination rates. The suboptimal uptake of COVID-19 and influenza vaccines, especially in those from socioeconomically deprived backgrounds and Black, mixed or other ethnic groups, underscores the necessity for interventions to reduce vaccine hesitancy and enhance acceptance in pregnant women.

## Introduction

Infections with COVID-19 and influenza during pregnancy can increase the risk of adverse pregnancy outcomes^1,2^ as pregnancy weakens the immune system^3^. Vaccinations against COVID-19^4^ and influenza^5^ were found to reduce these adverse outcomes and are therefore included in the routine immunisation schedule for pregnant women in the United Kingdom (UK)^6^. However, although COVID-19 and influenza vaccines are recommended for pregnant women, their uptake during pregnancy remains suboptimal^7,8^. This may be due to concerns about side effects and vaccine safety among pregnant women^9^, also known as vaccine hesitancy, which were found to be related to their demographics and baseline health conditions^7,10–12^.

Previous studies suggested the uptake of COVID-19 or influenza vaccines among pregnant women was lower in younger age groups^7,10^, black or unknown ethnicity^10,11^, deprived socioeconomic status^10,11^, those with no known risk factors for influenza^11,13^ and those living in large multigenerational household composition^12^. None of the available studies provided a population-level evaluation of COVID-19 and influenza vaccine uptake in pregnant women in England and Wales, particularly regarding how disparities in baseline characteristics impact uptake. Understanding vaccine uptake disparities in pregnant women would inform clinicians and policymakers in developing strategies to promote vaccination and reduce adverse pregnancy outcomes in the UK.

The uptake of COVID-19 and influenza vaccines in pregnant women during the COVID-19 pandemic could differ from normal times because of changes in vaccine hesitancy levels during a pandemic^14^ and the introduction and rollout of the new COVID-19 vaccines. Vaccine hesitancy may be more prevalent for COVID-19 vaccines due to limited evidence on maternal and neonatal safety available at the time of rollout^15,16^. The COVID-19 vaccination programme in the UK was initially rolled out centrally and followed a three-phase approach^17,18^, which may have had a significant impact on the timing of vaccination among pregnant women. Phase 1 of the rollout started on 8 December 2020, aiming to vaccinate the priority groups (health and care workers, those aged over 50, those considered clinically extremely vulnerable, and those aged over 16 with underlying health conditions) with two doses^18^. Phase 2 began in April 2021 and aimed to vaccinate people aged 18-49 with two doses^18^. Phase 3 included vaccinations for those aged 12 and over, as well as the rollout of booster vaccines starting in September 2021^18^. Influenza vaccination, on the other hand, was traditionally run through general practice in winter seasons, though pharmacies are also widely involved^19^.

We carried out this study to examine the COVID-19 and influenza vaccine uptake and disparities in pregnant women in England and Wales during the COVID-19 pandemic between September 2020 and March 2022. We described the characteristics of pregnant women eligible for COVID-19 and influenza vaccines in England and Wales. We assessed the uptake of COVID-19 and influenza vaccines in pregnant women during the pandemic. We investigated the COVID-19 and influenza vaccine uptake disparities in pregnant women by identifying associations between baseline characteristics (i.e., age, ethnicity, socioeconomic status, rurality, household size, obesity and the number of comorbidities) and receiving a vaccine.

## Results

### Cohort selection and baseline characteristics

A total of 133,300 pregnant women were eligible for COVID-19 vaccination during their pregnancy across England and Wales. We identified 178,690 pregnant women eligible for 2020/21 or 2021/22 seasonal influenza vaccination across England and Wales. There were 133,140 pregnant women in the study cohorts who were eligible for both COVID-19 and influenza vaccinations during pregnancy. The distribution of baseline characteristics was consistent between COVID-19 and influenza cohorts (Table 1).

**Table 1:** Descriptive characteristics for the COVID-19 and influenza cohorts across England and Wales

		COVID-19 vaccination eligible		Influenza vaccination eligible	
		Total	Vaccinated	Total	Vaccinated
Total		133,300	53,550	178,690	74,740
Age	18–24	23,320 (17.5%)	6380 (11.9%)	28,730 (16.1%)	9620 (12.9%)
	25–29	37,360 (28.0%)	13,250 (24.7%)	49,110 (27.5%)	20,480 (27.4%)
	30–34	42,400 (31.8%)	18,950 (35.4%)	58,320 (32.6%)	26,900 (36.0%)
	35–39	22,340 (16.8%)	10,830 (20.2%)	31,990 (17.9%)	14,250 (19.1%)
	40–49	7880 (5.9%)	4140 (7.7%)	10,540 (5.9%)	3490 (4.7%)
BMI	<18.5	5650 (4.2%)	1840 (3.4%)	7400 (4.1%)	2560 (3.4%)
	18.5-24.9	56,380 (42.3%)	23,540 (44.0%)	77,010 (43.1%)	32,860 (44.0%)
	25.0-29.9	33,030 (24.8%)	13,610 (25.4%)	44,730 (25.0%)	19,230 (25.7%)
	30.0-39.9	24,620 (18.5%)	9870 (18.4%)	32,610 (18.2%)	13,860 (18.5%)
	40.0+	5060 (3.8%)	1940 (3.6%)	5800 (3.2%)	2610 (3.5%)
	Unknown	8560 (6.4%)	2750 (5.1%)	11,160 (6.2%)	3610 (4.8%)
Ethnic groups	White	102,690 (77.0%)	42,960 (80.2%)	138,600 (77.6%)	60,870 (81.5%)
	Asian	11,300 (8.5%)	4550 (8.5%)	15,530 (8.7%)	6160 (8.2%)
	Black	5420 (4.1%)	1230 (2.3%)	6470 (3.6%)	1540 (2.1%)
	Mixed	2730 (2.0%)	900 (1.7%)	3540 (2.0%)	1140 (1.5%)
	Other	2410 (1.8%)	760 (1.4%)	3190 (1.8%)	1040 (1.4%)
	Unknown	8750 (6.6%)	3150 (5.9%)	11,370 (6.4%)	3980 (5.3%)
Household size	1	49,050 (36.8%)	17,870 (33.4%)	63,430 (35.5%)	24,380 (32.6%)
	2	50,100 (37.6%)	23,040 (43.0%)	69,880 (39.1%)	32,270 (43.2%)
	3	16,220 (12.2%)	6360 (11.9%)	21,710 (12.1%)	9010 (12.1%)
	4	9110 (6.8%)	3350 (6.3%)	12,090 (6.8%)	4790 (6.4%)
	5	4350 (3.3%)	1490 (2.8%)	5760 (3.2%)	2280 (3.1%)
	6–10	4010 (3.0%)	1270 (2.4%)	5260 (2.9%)	1860 (2.5%)
	11+	470 (0.4%)	160 (0.3%)	580 (0.3%)	150 (0.2%)
Socioeconomic status	1st (Most)	32,610 (24.5%)	9020 (16.8%)	41,150 (23.0%)	13,650 (18.3%)
	2nd	28,120 (21.1%)	10,180 (19.0%)	36,830 (20.6%)	14,280 (19.1%)
	3rd	25,390 (19.0%)	10,810 (20.2%)	34,610 (19.4%)	14,670 (19.6%)
	4th	24,690 (18.5%)	11,740 (21.9%)	34,110 (19.1%)	16,000 (21.4%)
	5th (Least)	22,490 (16.9%)	11,800 (22.0%)	32,000 (17.9%)	16,130 (21.6%)
Number of comorbidities	0	101,400 (76.1%)	41,290 (77.1%)	142,760 (79.9%)	58,710 (78.6%)
	1	26,900 (20.2%)	10,180 (19.0%)	30,310 (17.0%)	13,480 (18.0%)
	2	4230 (3.2%)	1720 (3.2%)	4720 (2.6%)	2110 (2.8%)
	3	620 (0.5%)	290 (0.5%)	720 (0.4%)	360 (0.5%)
	4+	160 (0.1%)	70 (0.1%)	170 (0.1%)	70 (0.1%)
Urban/rural area	Urban	110,140 (82.6%)	42,660 (79.7%)	146,300 (81.9%)	58,990 (78.9%)
	Rural	23,150 (17.4%)	10,890 (20.3%)	32,390 (18.1%)	15,740 (21.1%)

### Vaccine uptake in pregnant women

**Fig. 1:**
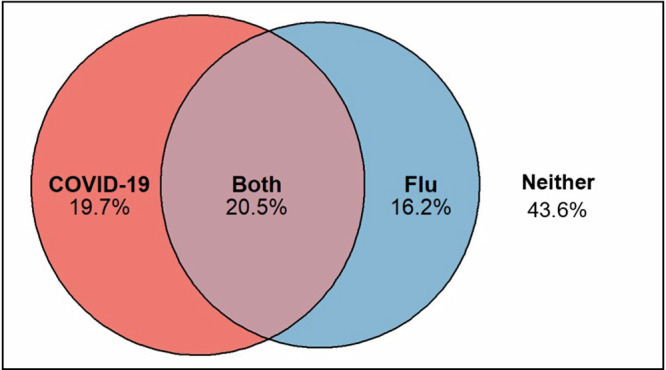
Overlap in vaccine uptake amongst those eligible for both vaccines during pregnancy in England and Wales (*N* = 133,140). The box shows all women eligible for both vaccines. The left circle shows those vaccinated for COVID-19. The right circle shows those vaccinated for influenza. The overlap of the two circles shows those who were vaccinated for both vaccines.

Of the 178,690 pregnant women in the influenza cohort, 74,740 (41.8%) received at least one dose of influenza vaccine (Table 1). Of the 133,300 individuals in the COVID-19 cohort, 53,550 (40.2%) received at least one dose of COVID-19 vaccine from any vaccination setting. Among the 133,140 pregnant women eligible for both vaccinations, 57,970 (43.6%) did not receive either vaccine, 27,350 (20.5%) received both vaccines, 26,190 (19.7%) only received the COVID-19 vaccine and 21,630 (16.2%) only received influenza vaccine (Fig. 1).

**Fig. 2:**
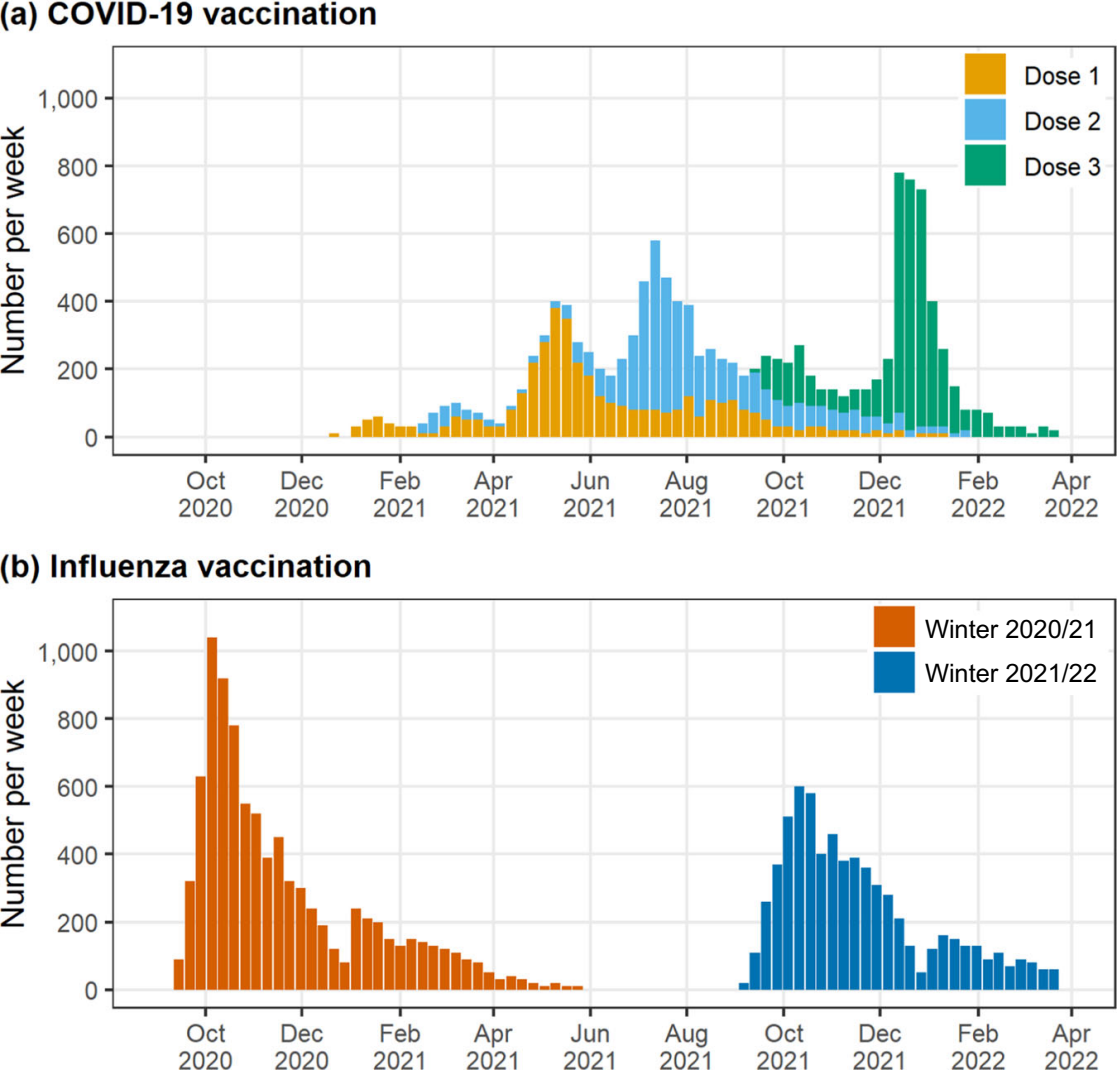
Vaccine uptake per week amongst pregnant women. **a** The weekly uptake of COVID-19 vaccination in pregnant women across England and Wales between September 2020 and March 2022, categorised by Dose 1, Dose 2 and Dose3. **b** The weekly uptake of influenza vaccination in pregnant women across England and Wales, categorised by the 2020/21 and 2021/22 influenza seasons.

Influenza vaccine uptake among pregnant women had a seasonal pattern and was highest between September and December in each season. The 2020/21 season saw a higher peak in the weekly number of influenza vaccinations than the 2021/22 season. The uptake of the COVID-19 vaccine in pregnant women was in line with the COVID-19 vaccination programme, with Phase 2 immunisation beginning in April 2021 (Fig. 2).

### Factors associated with vaccine uptake

**Fig. 3:**
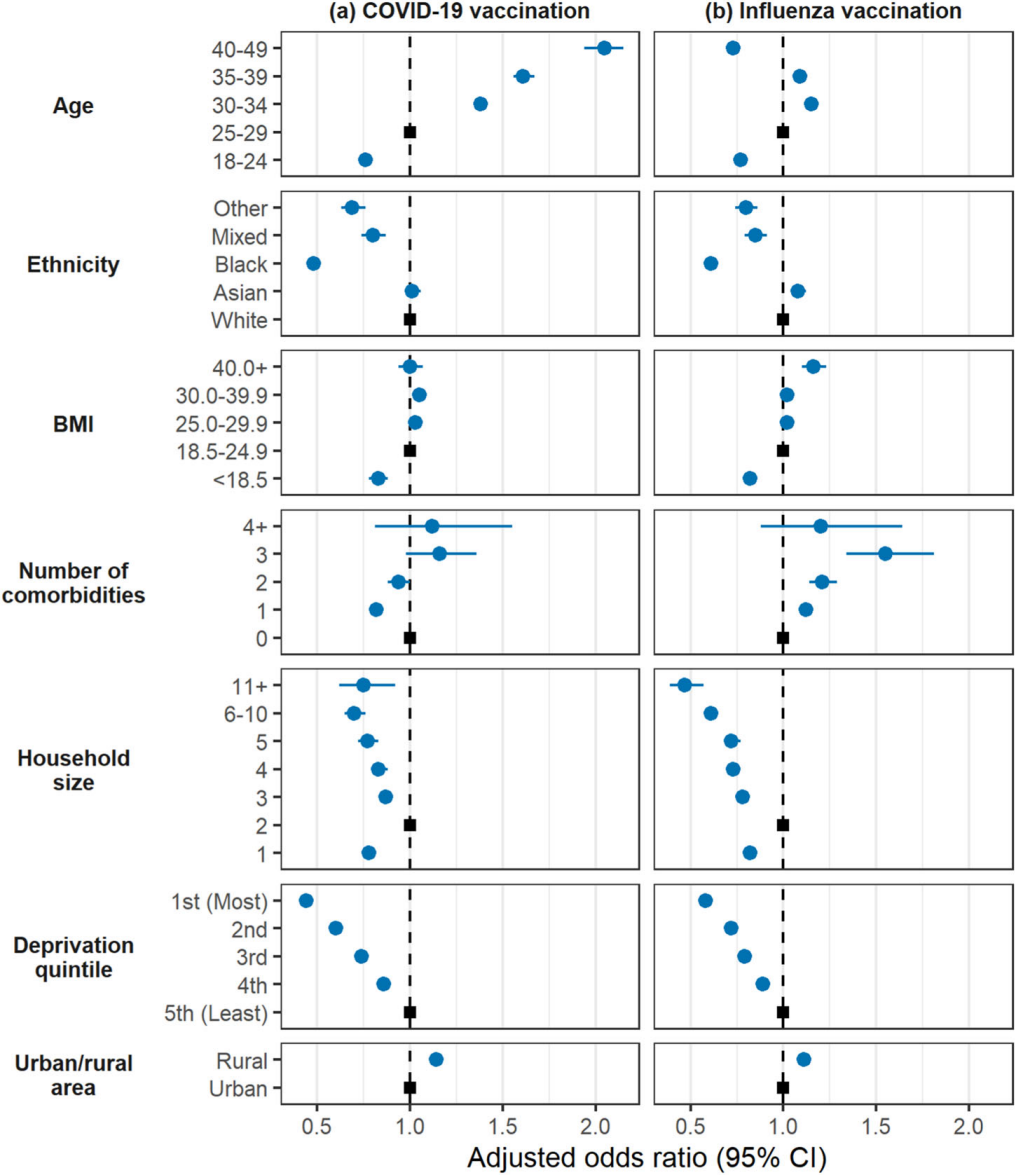
Factors associated with vaccine uptake in pregnant women. Factors associated with vaccine uptake for **a** COVID-19 (*N* =133,300) and **b** influenza (*N* = 178,690) in pregnant women in England and Wales between September 2020 and March 2022.

The COVID-19 and influenza vaccine uptake exhibited similar disparities across ethnic groups, deprivation quintile (i.e., socioeconomic status), household size, and rurality (Fig. 3). Pregnant women in the black ethnic group had the least chance of receiving either vaccine (COVID-19 aOR: 0.48, 95% Confidence Interval (CI): 0.45–0.51; Influenza aOR: 0.61, 95% CI: 0.57–0.65), while those of mixed (COVID-19 aOR: 0.80, 95% CI: 0.74–0.87; influenza aOR: 0.85, 95% CI: 0.79–0.91) and other (COVID-19 aOR: 0.69, 95%CI 0.63–0.76; influenza aOR: 0.80, 95% CI: 0.74–0.86) ethnic groups had a slightly higher chance of receiving vaccines, but still lower than those of white (reference group) or Asian ethnic group (COVID-19 aOR: 1.01, 95% CI: 0.97–1.06; influenza aOR: 1.08, 95% CI: 1.04–1.12).

There was a strong gradient of reduced vaccine uptake with the increase in deprivation. Pregnant women from the most deprived area had a much lower chance of receiving either vaccine (COVID-19 aOR: 0.44, 95% CI: 0.43–0.46; influenza aOR: 0.58, 95% CI: 0.56–0.60), compared to those from the least deprived areas. In comparison to households of two, vaccine uptake was lower in all the other household sizes. Additionally, those living in rural areas had a higher chance of receiving both vaccines than those living in urban areas (COVID-19 aOR: 1.14, 95% CI: 1.10–1.17; influenza aOR: 1.11, 95% CI: 1.08–1.14).

The vaccine uptake was inconsistent across age, comorbidity, and BMI groups between the two vaccines. Compared to the 25–29 age group, pregnant women aged 40–49 had the highest chance of receiving the COVID-19 vaccine (aOR: 2.05, 95% CI: 1.94–2.15), but the lowest chance of receiving the influenza vaccine (aOR: 0.73, 95% CI: 0.69–0.76). Meanwhile, pregnant women aged 18–24 had a low chance of receiving both vaccines (COVID-19 aOR: 0.76, 95% CI: 0.74–0.79; influenza aOR: 0.77, 95% CI: 0.75–0.80). Compared to pregnant women with no comorbidities, those with comorbidities had a higher chance of receiving the influenza vaccine. However, this trend was not observed for COVID-19 vaccines in pregnant women with one to two comorbidities. In comparison to pregnant women with normal BMI, pregnant women with a BMI over 40 (severely obese) had a higher chance of receiving influenza vaccine (aOR: 1.16, 95% CI: 1.10–1.23), while there was no difference in receiving COVID-19 vaccine (aOR: 1.00, 95%CI: 0.94–1.07). Pregnant women with a BMI under 18.5 had the lowest chance of receiving both vaccines (COVID-19 aOR: 0.83, 95% CI: 0.78–0.88, Influenza aOR: 0.82, 95% CI: 0.78–0.86).

## Discussion

Our analysis across England and Wales presented a low COVID-19 and influenza vaccine uptake among pregnant women during the COVID-19 pandemic, with COVID-19 vaccine uptake higher in 2021/22 and influenza vaccine uptake higher in the 2020/21 season. We identified vaccine uptake disparities across various baseline characteristics, particularly among different ethnic groups and socioeconomic statuses. Women of lower socioeconomic status had a significantly lower chance of receiving COVID-19 or influenza vaccination. Women in black, mixed, and other ethnic groups had a lower chance of being vaccinated in comparison to women in white or Asian ethnic groups.

The vaccine uptake in our study aligns with existing data. UKHSA estimated influenza vaccine uptake rates in pregnant women in England as 43.6% in 2020/21 and 37.9% in 2021/22^20^, which matches our observed rate of 41.8%. UKHSA reported that 32.3% of women who gave birth in England in September 2021 and 53.7% of women who gave birth in December 2021 had received at least one dose of COVID-19 vaccine^21,22^, aligning with our finding of 40.1%. The reports from UKHSA also support our observation that COVID-19 vaccine uptake was higher in the 2021/22 season, while influenza vaccine uptake was higher in the 2020/21 season among pregnant women.

Our study revealed disparities in COVID-19 and influenza vaccine uptake among pregnant women across various baseline characteristics during the COVID-19 pandemic. The determinants of accepting COVID-19 or influenza vaccines identified in our study include being socioeconomically affluent, of white or Asian ethnicity, living in rural areas, and residing in two-person households. These determinants align with findings from studies conducted in other countries, during non-pandemic periods, as well as from smaller-scale or qualitative studies^11,12,23–26^. We found that pregnant women aged 40–49 had a higher chance of receiving the COVID-19 vaccine but a lower chance of receiving the influenza vaccine. This finding was also noted in previous studies, where older age was identified as a predictor of receiving COVID-19 vaccines^7,27,28^ while being over 40 was linked to lower influenza vaccine uptake^11,24^.

The rollout strategy of the COVID-19 vaccine played an important role in vaccine uptake among pregnant women. The expansion of the influenza vaccination programme in the UK in 2020 aimed to safeguard vulnerable individuals from influenza, given the simultaneous circulation of COVID-19 and influenza viruses^29^. This initiative was important due to the limited availability of COVID-19 vaccines at the time^29^. The higher uptake of the influenza vaccine during the 2020/21 season, as found in our study, reflects the effect of the expanded influenza vaccination programme. Conversely, the increased uptake of the COVID-19 vaccine during the 2021/22 season reflects a more sufficient supply of COVID-19 vaccine. The difference in COVID-19 and influenza vaccine uptake among the 40–49 age group may also be relevant to the age-based rollout strategy for COVID-19 during the pandemic, as well as the heightened concerns associated with the novelty of the COVID-19 virus compared to influenza^30^.

Our study suggested that pregnant women with one or two comorbidities had a lower chance of accepting the COVID-19 vaccine compared to those with no comorbidities, which is opposite to the uptake pattern for the influenza vaccine. Influenza vaccine has been recommended for people in clinical risk groups (e.g. chronic respiratory disease, chronic heart disease and vascular disease, chronic kidney disease, etc.) in the UK since the 1960s^31,32^. The concept that the influenza vaccine can protect people with comorbidities from the risk of developing serious illness if they contract influenza has been well accepted in the general population^32,33^. Therefore, we observed pregnant women with one or two comorbidities had a higher chance of receiving influenza vaccine than those without comorbidities. In contrast, the safety of the COVID-19 vaccine in people with comorbidities was not fully studied at the time of the vaccination programme rollout^34^, which may increase vaccine hesitancy among patients with comorbidities^33^. The wide 95% confidence intervals shown for pregnant women with three or four comorbidities in the logistic regression results were mainly due to the small sample size in these two categories.

We found that the uptake for both COVID-19 and influenza vaccines was suboptimal in pregnant women during the COVID-19 pandemic, particularly among those in socioeconomically deprived groups and in black, mixed, and other ethnic groups. The mechanisms for lower vaccine uptake in people with more deprived socioeconomic status could include access to transport, confidence in vaccination, vaccination knowledge, and trust in healthcare or vaccination providers, according to an umbrella review^35^. A potential reason contributing to low vaccine uptake in ethnic minority groups could be a language barrier^36^. Another reason could be the over-registration in the UK primary care system. Over-registration is more people registered with a general practice than the estimated residents in the country. The over-registration rate in primary care in England was estimated to be 3.9% (*n* = 2,097,101) in 2014 and was found to be associated with non-White British residents and higher levels of social deprivation^37^. Pregnant women registered with a GP but not living in the UK are less likely to respond to the vaccine invitation, especially immigrants who choose to give birth in their home country. This issue can result in a falsely lower vaccine uptake among non-white ethnicities.

A strength of our study is that we used large national representative primary care data in England^38^ and Wales^39^. The longitudinal data provided insights into the trend of vaccination over seasons. Another strength is that we examined the vaccine uptake for both COVID-19 and influenza vaccines in the same population during the same time period. This study design facilitated a comparison between the vaccine uptake disparities for the two vaccines. Also, we adjusted the logistic regression model for multiple factors that may influence vaccine uptake decisions to minimise the risk of confounding and make the observed disparities in ethnicity and SES status more reliable.

There are limitations in this study. The influenza vaccine uptake information only accounted for vaccines recorded in the GP medical records and may underestimate the uptake rate. This is because vaccinations took place in pharmacies and other community or hospital settings, and vaccinations administered in other countries may not be recorded in the GP medical records. This may lead to bias in the results if certain groups of patients tend to receive the vaccine outside of general practices. Pregnancy in this study was identified from primary care medical records and may have delays in the recording of labour and miscarriages, which could cause misclassification of pregnant time periods. Also, the start date of pregnancy was derived using an algorithm based on the information available in the medical records, which may not be entirely accurate. The inclusion of non-term pregnancies in the study cohorts may introduce bias, as previous research has shown low COVID-19 vaccine uptake during the last trimester in Scotland^25^ and low influenza vaccine uptake during the first trimester in the UK^40^. Although our study adjusted for many factors, some factors, such as smoking status, educational background and changes in recommendations on COVID-19 vaccination for pregnant women, as well as other unmeasured confounding factors, were not accounted for in the analysis. Also, the granularity of ethnicity in our study was relatively broad, which may neglect the difference between ethnic minorities. For example, we grouped Bangladeshi/Pakistani people with Chinese people in the Asian ethnicity group, but the vaccine uptake hesitancy is much higher in Bangladeshi/Pakistani than in Chinese people^41^.

Our study emphasised the suboptimal uptake of COVID-19 and influenza vaccines among pregnant women, even during the COVID-19 pandemic when awareness of the importance of vaccination was heightened. Common reasons for vaccine hesitancy among pregnant women include fear of side effects or adverse events, lack of confidence in vaccine safety and low perceived risk of infection during pregnancy^9^. Although the safety of COVID-19 and influenza vaccines in pregnant women has been well proven in clinical studies^28,42^, it does not seem to be well perceived by the public. Pregnant women with one or two comorbidities showed particular concern about receiving COVID-19 vaccines. This concern could likely be alleviated by providing them with the most up-to-date evidence on COVID-19 vaccine safety.

Evidence shows that receiving a direct recommendation from healthcare providers, either through a consultation or a written message, can significantly increase influenza vaccine uptake in pregnant women^8,43^. Mitchell et al.^9^ constructed a framework that divided pregnant women into four distinctive groups according to their stage of vaccine hesitancy and recommended dedicated communication routes for each group^9^. Healthcare providers may use this framework to optimise their communication strategy, while more efforts should be put into ethnic minority and socioeconomically disadvantaged pregnant women. Frequent updates on new evidence regarding vaccine safety to healthcare providers and pregnant women are also recommended^44^.

In addition to the direct communication provided by healthcare providers, public agencies may routinely assess the efficacy and inequalities in vaccination delivery and implement policies to reduce such inequalities^17^. Our findings regarding vaccine uptake disparities are also informative for policy-making in vaccination programmes, particularly as vaccination against respiratory syncytial virus is under consideration for pregnant women by the UK Joint Committee on Vaccination and Immunisation^45^. On the research front, improved recording of vaccination information in electronic health records would be beneficial for future studies.

This study highlights the suboptimal uptake of COVID-19 and influenza vaccines in pregnant women during the COVID-19 pandemic, especially in those from socioeconomically deprived backgrounds and black, mixed or other ethnic groups. The COVID-19 phased rollout strategy had a strong impact on the COVID-19 and influenza vaccine uptake pattern in pregnant women during the pandemic. Disparities in COVID-19 and influenza vaccine uptake among pregnant women underscore the necessity for interventions from the perspectives of healthcare providers, public agencies, and scientists to reduce vaccine hesitancy and improve acceptance in pregnant women. Future studies may explore the reasons for vaccine uptake disparities identified in this study and investigate the relationship between receiving the COVID-19 vaccine and the influenza vaccine in pregnant women.

## Methods

### Data source

We used individual-level routinely collected primary care data and linked vaccine immunisation data from two separate large databases in England and Wales. For England, we used the nationally representative Oxford-Royal College of General Practitioners (RCGP) Research and Surveillance Centre (RSC) database, which covered around 32% of the English population (*N* > 19 million people) registered with over 1900 general practices across England^38^. The pseudonymised primary care data is linked to the National Immunisation Management Service for vaccination data. For Wales, we used the Secure Anonymised Information Linkage (SAIL) Databank trusted research environment (TRE), which covered 3·2 million people from 329 (84%) general practices across Wales^39^, linked to national COVID-19 vaccination data in Welsh Immunisation System^46^. Both the RCGP RSC database^38^ and the SAIL databank^39^ are primary care databases that are representative of both demographic and clinical aspects compared to the national population.

Primary care data provide pseudonymised information on patients’ demographics, disease diagnoses and some vaccinations recorded in general practices. Primary care services are the first point of contact in the UK healthcare system^47^, so primary care data linked with external vaccination databases would provide the complete patient demographics, medical and vaccination information needed for this study.

In England, ethical approval was granted by the Health Research Authority London Central Research Ethics Committee (reference number REC reference 21/HRA/2786; integrated research application system number 30174). In Wales, research conducted within the Secure Anonymised Information Linkage Databank was done with the permission and approval of the independent Information Governance Review Panel (project number 0911). Individual written patient consent was not required for this study.

### Cohort selection

**Fig. 4:**
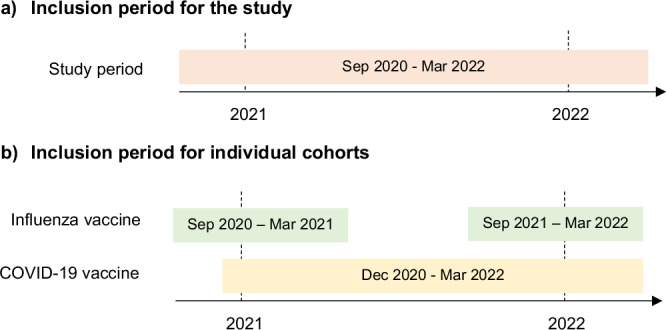
Patient inclusion periods. **a** The inclusion period for this study starts in September 2020 and ends in March 2022. **b** The inclusion period for the influenza vaccine was divided into two seasons: the 2020/21 season, which covered 1 September 2020–31 March 2021, and the 2021/22 season, which covered 1 September 2021–31 March 2022. The inclusion period for the COVID-19 vaccine started on 8 December 2020 and ended on 31 March 2022.

The two national cohorts included women aged 18 to 49 who were eligible for either COVID-19 vaccination, influenza vaccination or both during the course of pregnancy between September 2020 and March 2022 in England and Wales (Fig. 4).

In the UK, pregnant women have been eligible for free influenza vaccination in the influenza season since 2010 ^48^. For the present study, we analysed influenza vaccinations during the 2020/21 and 2021/22 seasons. Eligibility for the COVID-19 vaccination for pregnant women changed over time. Pregnant women were first offered the COVID-19 vaccine in December 2020 (Phase 1 rollout) if they were health and social care workers or in an at-risk group^49^. They were then eligible based on age groups from April 2021 (Phase 2 rollout) and were added to the clinical risk groups for COVID-19 vaccination in December 2021^49^. Thus, analysis for COVID-19 and influenza vaccines was performed on two separate cohorts to account for differences in these vaccination programmes (Fig. 4).

The influenza cohort included pregnant women who were pregnant for at least 30 days during the seasonal influenza immunisation programme rollout period in either the 2020/21 season (1 September 2020–31 March 2021) or the 2021/22 season (1 September 2021–31 March 2022).

The COVID-19 cohort comprised women who were pregnant for at least 30 days after they were eligible to receive a COVID-19 vaccination, whether first or any subsequent dose between 8 December 2020 and 31 March 2022. This means the women must have either been unvaccinated 30 days into their pregnancy and become eligible at least 30 days before the end of pregnancy or if they have already had a vaccination (dose 1 or 2) before 30 days into pregnancy, they must have become eligible for a second or third dose (over 8 weeks after the previous dose) at latest 30 days before the end of pregnancy.

The start date of a pregnancy was defined as the first day of the last menstrual period. The end date of pregnancy was defined as the date of the delivery of the foetus or the termination of the pregnancy, such as miscarriage. Both dates were identified from the primary care medical records using a developed algorithm^50,51^. For pregnant women who had more than one pregnancy during the study period, we included only their first pregnancy for analysis.

### Outcome measure

Our primary outcomes were the uptake of the COVID-19 and the influenza vaccine during pregnancy between September 2020 and March 2022. The vaccine uptake was defined as the number of pregnant women in the cohort who received at least one vaccination during the study period, divided by the total number of pregnant women in the cohort, and presented as a percentage.

Baseline characteristics were measured as predicting factors (i.e. independent variable) for vaccination. We measured age (categorised as 18–24, 25–29, 30–34, 35–39 and 40–49), ethnic groups (categorised as white, Asian, black, mixed, others and unknown), body mass index (BMI, categorised as <18.5, 18.5–24.9, 25.0–29.9, 30.0–39.9, 40.0 or more and unknown), number of comorbidities (categorised as none, 1, 2, 3 and 4 or more), household size (categorised as 1, 2, 3, 4, 5, 6–10 and 11 or more), socioeconomic status of the residential area (categorised as 1—most deprived, 2, 3, 4 and 5—least deprived) as well as rurality. Additionally, the residing health board for Wales (e.g. Aneurin Bevan University Health Board, Betsi Cadwaladr University Health Board, Cardiff and Vale University Health Board, etc.) and regions for England (e.g. London, the North West, North East, Yorkshire, East Midlands, etc.) were included to control for potential confounding effects caused by differences in the distribution of the population as well as delivery of the vaccination programmes within each country.

Data for these factors are available from the primary care databases RCGP and SAIL Databank. Demographics (i.e. age, ethnicity, BMI, household size and socioeconomic status) were identified at the study start date. The traceback period for identifying comorbidities was five years. Socioeconomic status was based on the quintiles of the 2015 Index of Multiple Deprivation (IMD) in England and^52^ the 2019 Welsh Index of Multiple Deprivation (WIMD) in Wales^53^ and was derived using patients’ Lower-layer Super Output Area (LSOA) of residence. Since BMI and ethnic groups were not available for all the individuals, we included an unknown category to represent this. Household size was determined by the number of family members registered at the same GP practice as the pregnant women.

The comorbidities in this study were defined in line with the clinical risk groups for the COVID-19 immunisation programme as stated in Chapter 14a in Immunisation Against Infectious Disease (The Green Book)^49^, the UK immunisation guidance. The comorbidities included are chronic respiratory disease, chronic heart disease, chronic renal disease, chronic liver disease, chronic neurological disease, diabetes mellitus and immunosuppression. For England, these comorbidities were identified as part of the PRIMIS v2.3 specification^54^, a national data specification commissioned by the UK Health Security Agent (UKHSA) to help identify priority patients for the COVID-19 vaccination. For Wales, the comorbidities were derived from QCOVID indicators^55^ that were part of a COVID-19 risk prediction model.

### Statistical analysis

We used descriptive analysis to present the baseline demographics and comorbidities of the two cohorts (i.e. the COVID-19 cohort and the influenza cohort). We presented the weekly number of vaccinations received by individuals in each cohort over the study period in bar charts. We also presented the vaccine uptake in the sub-cohort of women eligible for both vaccines during their pregnancy, which included uptake of influenza vaccine only, uptake of COVID-19 vaccine only, uptake of both vaccines and uptake of neither vaccine.

We conducted a multivariable fixed-effect logistic regression analysis to explore factors associated with vaccine uptake among pregnant women. The regression estimates adjusted odds ratios (aOR) for the covariates, which were reported with 95% confidence intervals (95% CIs).

Descriptive statistics (frequencies) were summed, and the cohort-specific log odds ratios were meta-analysed to produce summary odd ratios. A fixed effect model was used as the same effects were anticipated in each country and as we are only using meta-analysis methods to replicate a pooled analysis. All analytical work was done using R version 4.1.3^56^.

### Supplementary information

Supplementary Information

## Data Availability

The data that support the findings of this study are available from Oxford-Royal College of General Practitioners (RCGP) Research and Surveillance Centre (RSC) database and the Secure Anonymised Information Linkage (SAIL) Databank trusted research environment (TRE) but restrictions apply to the availability of these data, which were used under licence for the current study, and so are not publicly available. Data are, however, available from the authors upon reasonable request and with permission of RCGP RSC and SAIL TRE.

## Code availability

The code used for data analysis and processing in this manuscript is available upon request from the corresponding author.
