# Supplementary material for: COVID-19 and influenza vaccine uptake among pregnant women in national cohorts of England and Wales
Source: NPJ Vaccines. 2024 Aug 14;9:147. doi: 10.1038/s41541-024-00934-9 (PMC11324884; doi:10.1038/s41541-024-00934-9)
Supplement: Supplementary file 1 — Supplementary Information [file 41541_2024_934_MOESM1_ESM.pdf]

Supplementary Table 1: Descriptive characteristics for the COVID-19 and influenza cohorts in England and Wales.

(a) England-only results

		**COVID-19 vaccination eligible**		**Influenza vaccination eligible**	
		**Total**	**Vaccinated**	**Total**	**Vaccinated**
**Total**		111,010	44,750	146,980	59,170
**Age**	18-24	18,590(16.7%)	4,950(11.1%)	22,340(15.2%)	6,800(11.5%)
	25-29	30,250(27.2%)	10,590(23.7%)	39,100(26.6%)	15,670(26.5%)
	30-34	35,450(31.9%)	15,850(35.4%)	48,240(32.8%)	21,660(36.6%)
	35-39	19,350(17.4%)	9,440(21.1%)	27,510(18.7%)	11,920(20.1%)
	40-49	7,370(6.6%)	3,920(8.8%)	9,790(6.7%)	3,120(5.3%)
**BMI**	<18.5	4,920(4.4%)	1,620(3.6%)	6,340(4.3%)	2,150(3.6%)
	18.5-24.9	47,980(43.2%)	20,190(45.1%)	64,850(44.1%)	26,900(45.5%)
	25.0-29.9	26,430(23.8%)	10,960(24.5%)	35,310(24.0%)	14,570(24.6%)
	30.0-39.9	19,070(17.2%)	7,640(17.1%)	24,730(16.8%)	9,940(16.8%)
	40.0+	4,050(3.6%)	1,600(3.6%)	4,600(3.1%)	2,000(3.4%)
	Unkown	8,560(7.7%)	2,750(6.1%)	11,160(7.6%)	3,610(6.1%)
**Ethnic groups**	White	81,920(73.8%)	34,720(77.6%)	109,150(74.3%)	46,330(78.3%)
	Asian	10,580(9.5%)	4,250(9.5%)	14,470(9.8%)	5,640(9.5%)
	Black	5,230(4.7%)	1,180(2.6%)	6,190(4.2%)	1,420(2.4%)
	Mixed	2,470(2.2%)	820(1.8%)	3,160(2.1%)	990(1.7%)
	Other	2,140(1.9%)	660(1.5%)	2,760(1.9%)	850(1.4%)
	Unkown	8,670(7.8%)	3,130(7.0%)	11,250(7.7%)	3,940(6.7%)
**Household size**	1	47,450(42.7%)	17,210(38.5%)	61,020(41.5%)	23,110(39.1%)
	2	45,170(40.7%)	20,930(46.8%)	62,640(42.6%)	28,350(47.9%)
	3	9,660(8.7%)	3,660(8.2%)	12,360(8.4%)	4,240(7.2%)
	4	4,810(4.3%)	1,730(3.9%)	6,110(4.2%)	2,010(3.4%)
	5	1,970(1.8%)	630(1.4%)	2,460(1.7%)	790(1.3%)
	6-10	1,630(1.5%)	470(1.1%)	2,020(1.4%)	580(1.0%)
	11+	330(0.3%)	120(0.3%)	390(0.3%)	90(0.2%)
**Socioeconomic status**	1st (Most)	26,650(24.0%)	7,180(16.0%)	32,840(22.3%)	10,150(17.2%)
	2nd	23,260(21.0%)	8,400(18.8%)	30,010(20.4%)	11,070(18.7%)
	3rd	21,270(19.2%)	9,100(20.3%)	28,680(19.5%)	11,640(19.7%)
	4th	20,880(18.8%)	10,040(22.4%)	28,640(19.5%)	13,050(22.1%)
	5th (Least)	18,950(17.1%)	10,040(22.4%)	26,820(18.2%)	13,250(22.4%)
**Number of comorbidities**	0	84,590(76.2%)	34,310(76.7%)	117,260(79.8%)	46,280(78.2%)
	1	21,540(19.4%)	8,410(18.8%)	24,240(16.5%)	10,420(17.6%)
	2	4,120(3.7%)	1,680(3.8%)	4,590(3.1%)	2,040(3.4%)
	3	610(0.5%)	280(0.6%)	710(0.5%)	350(0.6%)
	4+	160(0.1%)	70(0.2%)	170(0.1%)	70(0.1%)
**Urban/rural area**	Urban	93,670(84.4%)	36,260(81.0%)	122,850(83.6%)	47,700(80.6%)
	Rural	17,340(15.6%)	8,490(19.0%)	24,130(16.4%)	11,470(19.4%)

(b) Wales-only results

		COVID-19 vaccination eligible		Influenza vaccination eligible	
		**Total**	**Vaccinated**	**Total**	**Vaccinated**
**Total**		22,290	8,790	31,720	15,560
**Age**	18-24	4,730(21.2%)	1,430(16.3%)	6,390(20.1%)	2,820(18.1%)
	25-29	7,110(31.9%)	2,660(30.3%)	10,010(31.6%)	4,810(30.9%)
	30-34	6,950(31.2%)	3,100(35.3%)	10,080(31.8%)	5,240(33.7%)
	35-39	2,990(13.4%)	1,390(15.8%)	4,480(14.1%)	2,330(15.0%)
	40-49	510(2.3%)	220(2.5%)	750(2.4%)	370(2.4%)
**BMI**	<18.5	730(3.3%)	220(2.5%)	1,060(3.3%)	410(2.6%)
	18.5-24.9	8,400(37.7%)	3,350(38.1%)	12,160(38.3%)	5,960(38.3%)
	25.0-29.9	6,600(29.6%)	2,650(30.1%)	9,420(29.7%)	4,660(29.9%)
	30.0-39.9	5,550(24.9%)	2,230(25.4%)	7,880(24.8%)	3,920(25.2%)
	40.0+	1,010(4.5%)	340(3.9%)	1,200(3.8%)	610(3.9%)
	Unkown	0(0.0%)	0(0.0%)	0(0.0%)	0(0.0%)
**Ethnic groups**	White	20,770(93.2%)	8,240(93.7%)	29,450(92.8%)	14,540(93.4%)
	Asian	720(3.2%)	300(3.4%)	1,060(3.3%)	520(3.3%)
	Black	190(0.9%)	50(0.6%)	280(0.9%)	120(0.8%)
	Mixed	260(1.2%)	80(0.9%)	380(1.2%)	150(1.0%)
	Other	270(1.2%)	100(1.1%)	430(1.4%)	190(1.2%)
	Unkown	80(0.4%)	20(0.2%)	120(0.4%)	40(0.3%)
**Household size**	1	1,600(7.2%)	660(7.5%)	2,410(7.6%)	1,270(8.2%)
	2	4,930(22.1%)	2,110(24.0%)	7,240(22.8%)	3,920(25.2%)
	3	6,560(29.4%)	2,700(30.7%)	9,350(29.5%)	4,770(30.7%)
	4	4,300(19.3%)	1,620(18.4%)	5,980(18.9%)	2,780(17.9%)
	5	2,380(10.7%)	860(9.8%)	3,300(10.4%)	1,490(9.6%)
	6-10	2,380(10.7%)	800(9.1%)	3,240(10.2%)	1,280(8.2%)
	11+	140(0.6%)	40(0.5%)	190(0.6%)	60(0.4%)
**Socioeconomic status**	1st (Most)	5,960(26.7%)	1,840(20.9%)	8,310(26.2%)	3,500(22.5%)
	2nd	4,860(21.8%)	1,780(20.3%)	6,820(21.5%)	3,210(20.6%)
	3rd	4,120(18.5%)	1,710(19.5%)	5,930(18.7%)	3,030(19.5%)
	4th	3,810(17.1%)	1,700(19.3%)	5,470(17.2%)	2,950(19.0%)
	5th (Least)	3,540(15.9%)	1,760(20.0%)	5,180(16.3%)	2,880(18.5%)
**Number of comorbidities**	0	16,810(75.4%)	6,980(79.4%)	25,500(80.4%)	12,430(79.9%)
	1	5,360(24.0%)	1,770(20.1%)	6,070(19.1%)	3,060(19.7%)
	2	110(0.5%)	40(0.5%)	130(0.4%)	70(0.4%)
	3	10(0.0%)	10(0.1%)	10(0.0%)	10(0.1%)
	4+	0(0.0%)	0(0.0%)	0(0.0%)	0(0.0%)
**Urban/rural area**	Urban	16,470(73.9%)	6,400(72.8%)	23,450(73.9%)	11,290(72.6%)
	Rural	5,810(26.1%)	2,400(27.3%)	8,260(26.0%)	4,270(27.4%)
